# Interaction-Based Feature Selection Algorithm Outperforms Polygenic Risk Score in Predicting Parkinson’s Disease Status

**DOI:** 10.3389/fgene.2021.744557

**Published:** 2021-10-20

**Authors:** Justin L. Cope, Hannes A. Baukmann, Jörn E. Klinger, Charles N. J. Ravarani, Erwin P. Böttinger, Stefan Konigorski, Marco F. Schmidt

**Affiliations:** ^1^ biotx.ai GmbH, Potsdam, Germany; ^2^ Digital Health Center, Hasso Plattner Institute for Digital Engineering, University of Potsdam, Potsdam, Germany

**Keywords:** epistasis, machine learning, feature selection, parkinson's disease, PPMI (parkinson’s progression markers initiative)

## Abstract

Polygenic risk scores (PRS) aggregating results from genome-wide association studies are the state of the art in the prediction of susceptibility to complex traits or diseases, yet their predictive performance is limited for various reasons, not least of which is their failure to incorporate the effects of gene-gene interactions. Novel machine learning algorithms that use large amounts of data promise to find gene-gene interactions in order to build models with better predictive performance than PRS. Here, we present a data preprocessing step by using data-mining of contextual information to reduce the number of features, enabling machine learning algorithms to identify gene-gene interactions. We applied our approach to the Parkinson’s Progression Markers Initiative (PPMI) dataset, an observational clinical study of 471 genotyped subjects (368 cases and 152 controls). With an AUC of 0.85 (95% CI = [0.72; 0.96]), the interaction-based prediction model outperforms the PRS (AUC of 0.58 (95% CI = [0.42; 0.81])). Furthermore, feature importance analysis of the model provided insights into the mechanism of Parkinson’s disease. For instance, the model revealed an interaction of previously described drug target candidate genes *TMEM175* and *GAPDHP25*. These results demonstrate that interaction-based machine learning models can improve genetic prediction models and might provide an answer to the missing heritability problem.

## Introduction

The need to understand how to predict phenotypes from genetic data is becoming ever-more important for the prediction of disease risk for individuals and for plant and animal breeding as well as for genome editing. Polygenic risk scores (PRS), simple additive models, are the state of the art in the investigation of the genetic architecture of complex traits or diseases, and, more importantly, in the prediction of disease susceptibility. (Wray et al., 2007; Evans et al., 2009; International Schizophrenia Consortium et al., 2009). A Polygenic Risk Score is calculated for a given individual as the weighted sum of the number of risk allele single nucleotide polymorphisms (SNP) for which the individual was tested. The weights used in this calculation are the regression coefficients from a prior genome-wide association study (GWAS).

Importantly, PRS models are not optimized for predictive performance. (Chatterjee et al., 2013; Dudbridge, 2013). There are three reasons for this:

(1) Due to the current limited sample size of discovery GWAS datasets (<1,000,000 individuals), biologically relevant rare variants with small effect sizes cannot be detected. Additionally, the limited sample sizes of discovery GWAS can lead to biased PRS models that might not perform well in populations with ancestry different to that of the discovery dataset. (Reisberg et al., 2017; Duncan et al., 2019).
(2) It has been shown that statistically significant outcome-associated SNPs are not automatically good predictors of that outcome. (Lo et al., 2015).
(3) It has been reported that genetic effects discovered in genome-wide association studies do not sum to the estimate of the heritability of the trait derived from twin studies. (Yang et al., 2010). This has been called the *missing heritability problem* in GWAS. (Manolio et al., 2009). Besides potentially missing relevant rare variants and suboptimal SNP selection based on *p*-values, classical PRS models ignore complex gene-gene interactions, also known as *epistasis*, of the trait or disease due to their simple additive structure.

The concept of epistasis was first described more than 100 years ago. (Bateson, 1906). Statistical epistasis, as observed in genome-wide association studies, is genetic variance that can be attributed to gene interaction and is defined as a function of the allele frequencies in a population. Detection of epistasis in discovery GWAS and modeling its impact is challenging because of linkage disequilibrium (LD), replication of identified gene-gene interactions in validation datasets, model complexity, and high dimensionality. (Wei et al., 2014).

Machine learning algorithms that improve automatically through the use of data represent an opportunity to find gene-gene interactions in order to build models with better predictive performance than PRS. Nevertheless, in a recent study, a PRS model outperformed five machine learning algorithms (Naïve Bayes classifier, regularized regression, random forest, gradient boosting, and support vector machine) that were used to build predictive models for coronary artery disease status. (Gola et al., 2020).

Here we revisit the potential of machine learning algorithms to predict disease status compared to a PRS model. For this purpose, we adopt the Parkinson’s Progression Markers Initiative (PPMI) dataset (Marek et al., 2011, 2018) (https://www.ppmi-info.org) as this dataset has been intensively analyzed and is broadly available for replication studies. We explore two machine learning approaches in particular, which complement those applied by Gola et al.: deep learning and interaction-based feature selection. The first approach, deep learning, employs artificial neural networks to discover automatically from raw data the representations needed for classification. Despite not being widely used in the field of genomics, there is work on applying deep learning to GWAS: Romero et al., 2016 predict genetic ancestry by introducing a multi-task architecture including a parameter prediction network, thereby considerably reducing the feature space under consideration. The second approach, interaction-based feature selection, also drastically reduces the feature space–in this case, by leveraging contextual information obtained via data mining, allowing for the testing of a small set of complex hypotheses containing interactions of multiple variants. Further details concerning these approaches are described in the Methods section, following a presentation of the results of our investigation below.

## Results

### Data Preparation

For all 471 subjects in the PPMI database (368 cases and 152 controls) subject genotyping information was collected from two complementary genotyping chips (NeuroX and ImmunoChip). After careful quality control and harmonization, we merged that information into a single dataset with 369,036 variants and 436 individuals (296 cases and 140 controls). The data was then split into three disjoint sets: a training set (*n* = 367) for training predictive models; a validation set (*n* = 33) for so-called *hyperparameter tuning*, and a test set (*n* = 36) for model evaluation. Training and validation are described in further detail below for each approach as appropriate. In all cases, evaluation metrics were calculated on the basis of bootstrap resampling with 10^4^ iterations.

### Genome-Wide Association Study

**FIGURE 1:**
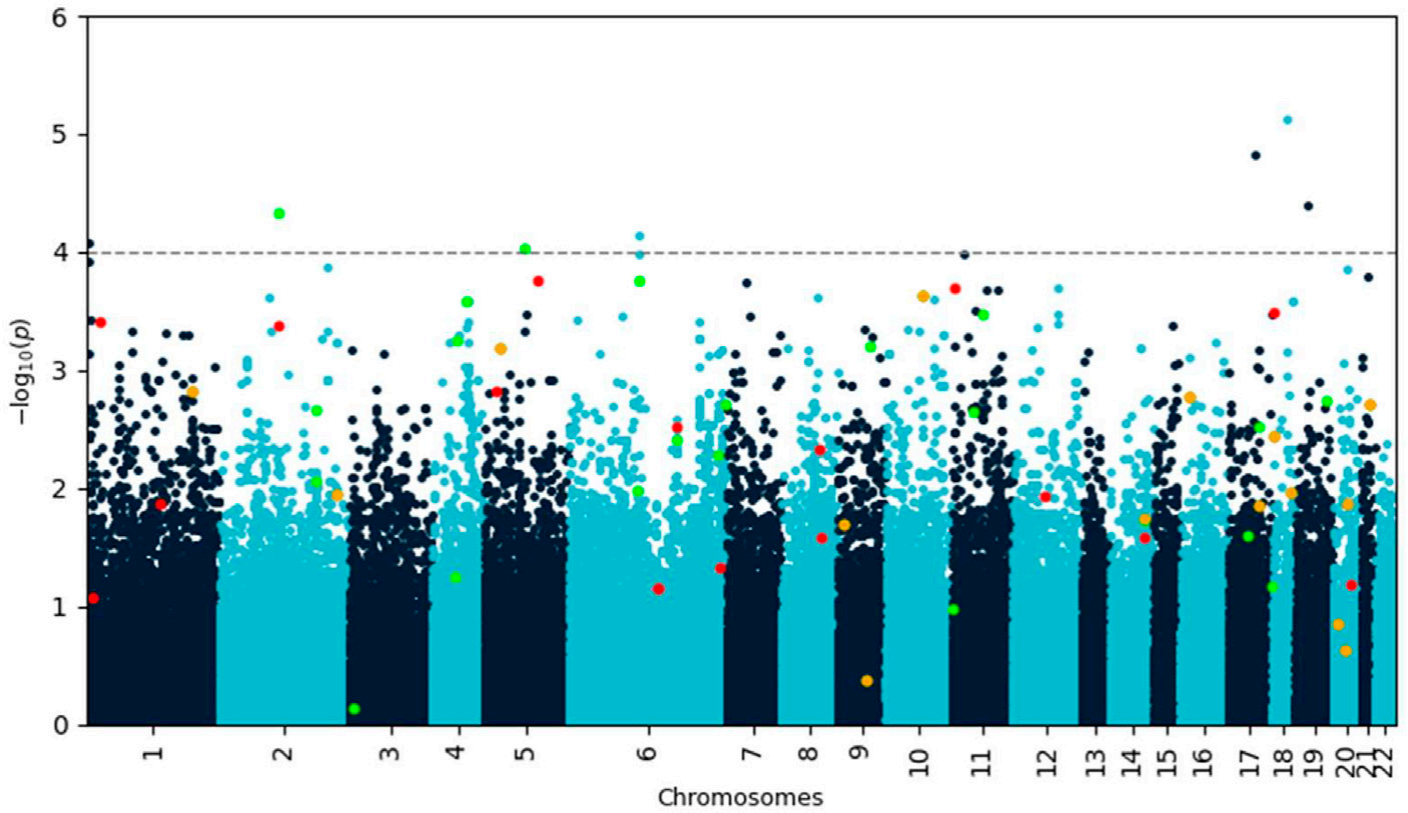
Manhattan plot of negative decadic logarithm of *p*-values for SNPs as determined by SAIGE analysis. Variants identified by Lasso with feature selection are highlighted in red and green if they increase or decrease disease risk, respectively. Variants highlighted in orange occur in both protective and risk-enhancing groups of SNPs, depending on their genotype. Most of these biologically meaningful variants would have been missed by using a simple *p*-value cutoff.

A genome-wide association (GWA) analysis was performed on the training data. The Manhattan plot of the *p*-values resulting from the analysis is shown in Figure 1. Seven single nucleotide polymorphisms (SNPs) showed *p*-values less than 10^–4^ (Table 1).

**TABLE 1:** PPMI GWAS results identified seven SNPs with a *p*-value < 10^–4^. Positions and rs IDs according to Human Genome Reference hg19 (GRCh37).

Chr	Pos	SNP Id	rs Id	Gene	*p*-value
1	173,266,578	imm_1_171,533,201	rs4916319	*TNFSF4* (upstream)	0.000083
2	209,087,335	exm2261159	rs4675743		0.000046
5	156,376,703	exm498917	rs6873053	*TIMD4* (downstream)	0.000092
6	133,716,974	rs212805	rs212805	*EYA4*	0.000074
17	25,895,033	imm_17_22,919,160	rs4795747		0.000015
18	5,479,093	rs7238186	rs7238186	*EPB41L3* (downstream)	0.000007
19	57,909,872	exm1513284	rs4801478	*ZNF548*	0.000040

### Polygenic Risk Score

To calculate the PRS, seven different *p*-value thresholds (0.001, 0.05, 0.1, 0.2, 0.4, and 0.5) for the subjects in the training, validation and test set were used. The PRS of the subjects in the training set were then used to train a separate logistic regression classifier for each *p*-value threshold. Receiver operating characteristics (ROC) curves were used to evaluate classifier performance relative to the validation data. The classifier with the highest mean area under the curve (AUC) was that which had been trained on the PRS resulting from the 0.05 *p*-value threshold, comprising the weighted sum of 57 different SNPs. This classifier was finally evaluated relative to the test data set, where the mean AUC was 0.58 with a 95% confidence interval from 0.42 to 0.81 Figure 3. Table 2 presents these results, along with the estimates of accuracy, sensitivity, and specificity corresponding to the optimal Youden’s index of 0.21.

**FIGURE 2:**
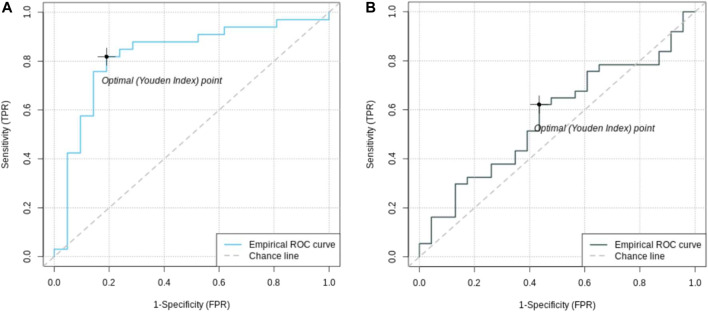
Receiver operating characteristic (ROC) curves of feature selected machine learning model **(A)** and polygenic risk score **(B)**. The AUC of the feature selected model with 0.85 [95% CI = (0.72; 0.96)] is better than the AUC of the PRS with 0.56 [95% CI = (0.42; 0.81)].

**FIGURE 3:**
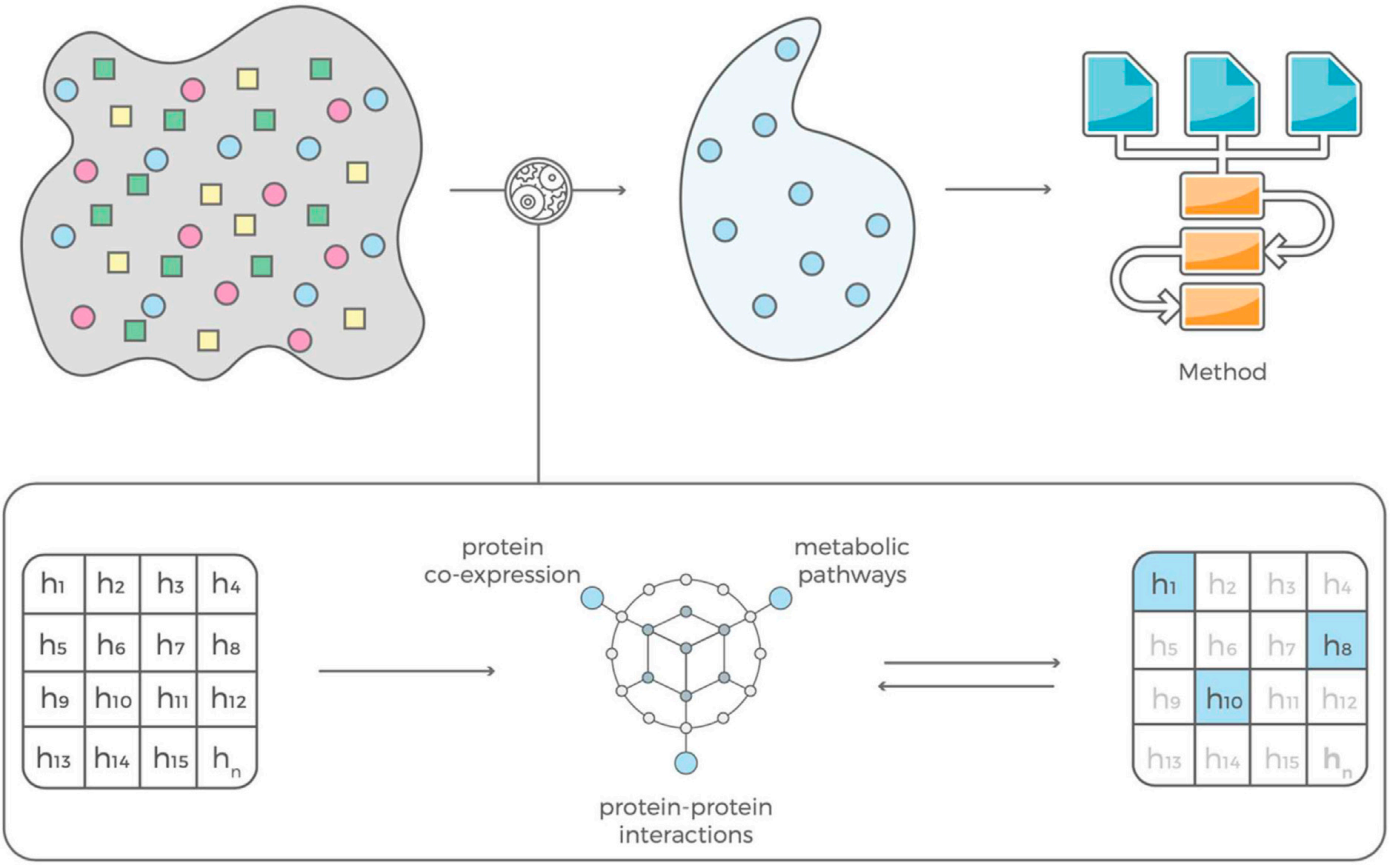
Our feature selection consists of two complementary modules that are in feedback with each other. The contextual module uses information mined from the scientific literature, pathway libraries and protein co-expression data and an evaluation module that estimates predictive power of a feature based on that contextual information. The selected features can be used to build prediction models with standard machine learning algorithms.

**TABLE 2:** Performance comparison of all models.

Method	AUC [95% CI]	Accuracy	Sensitivity	Specificity	Youden’s index
PRS	0.56 [0.42; 0.81]	0.60	0.62	0.56	0.21
Deep learning	0.67 [0.47; 0.83]	0.60	0.42	0.88	0.29
LASSO w/feature selection	0.85 [0.72; 0.96]	0.81	0.81	0.80	0.61
LASSO w/o feature selection	0.51 [0.39; 0.63]	0.62	0.87	0.09	0.12

### Deep Learning

We applied Romero *et al.*‘s approach to the PPMI dataset, again using the training data set to train competing networks with distinct hyperparameter settings and the validation data set to select between these networks. When evaluated relative to the test data set, the mean AUC of the final deep learning model was 0.67 (95% CI = [0.47; 0.83]) and the optimal Youden index corresponding to the accuracy, sensitivity, and specificity measures reported in Table 2 was 0.29.

### Feature Selection and LASSO Regression

**FIGURE 4:**
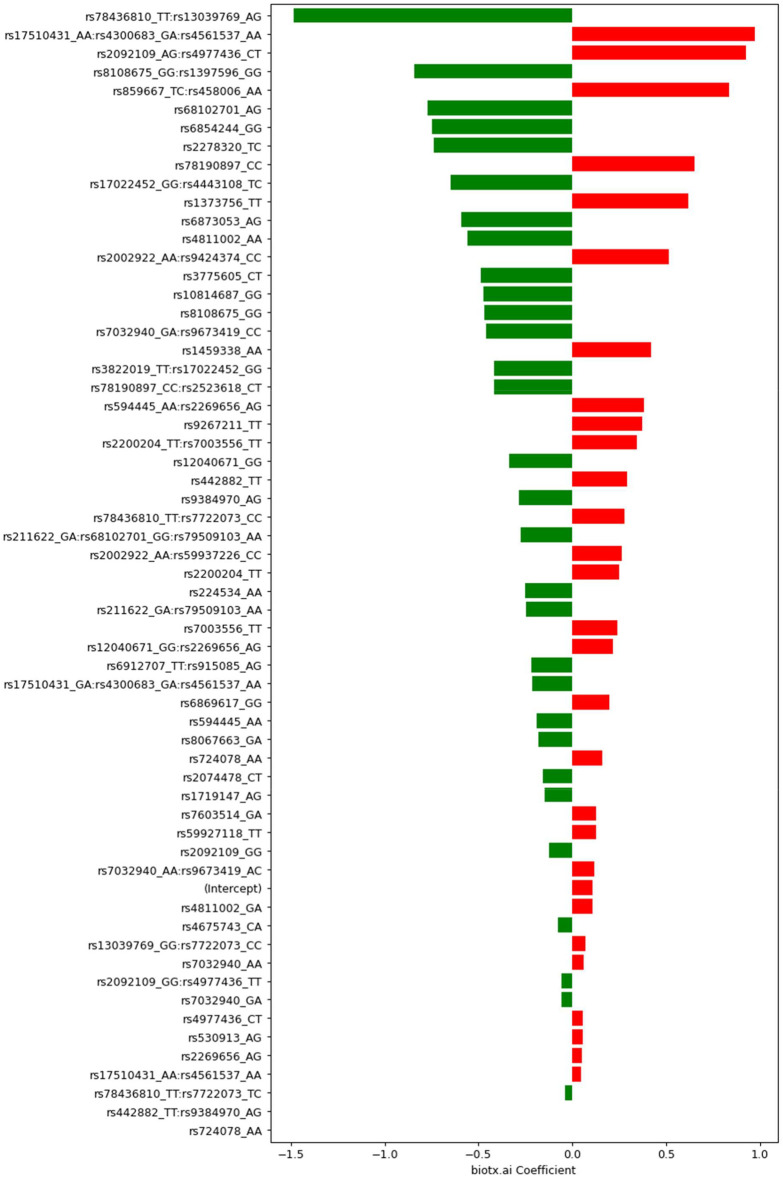
Coefficients determined by Lasso with feature selection for SNPs and groups of SNPs. Negative values (green) indicate protective (combinations of) variants, positive values (red) mark risk variants. The respective genotypes of each variant are indicated by one-letter codes of the bases, where the first letter corresponds to the reference allele, and the second corresponds to the observed, alternative allele.

A set of less than 100 polygenic hypotheses were generated using the interaction-based feature selection approach applied to the training data, as described in the Methods section below. (See also an overview of our approach in Figure 3.). These hypotheses were summarized in a term that was used to build a LASSO regression model on the basis of the validation data. (Tibshirani, 1996). The predictive performance of this model, based on 47 SNPs in several different interaction terms, was then evaluated relative to the test set Figure 4. The mean area under the curve (AUC) for the LASSO model with prior feature selection was 0.85 [95% CI = (0.72; 0.96)] and the optimal Youden index corresponding to the accuracy, sensitivity, and specificity measures reported in Table 2 was 0.61. A LASSO model without prior feature selection that was built for comparison was evaluated in the same manner but did not deliver outcomes that were significantly better than chance (Table 2), in line with Gola et al. (2020) s results for regularized regression.

Exploring the feature selection based model with its interactive terms provides insights about the genes associated with Parkinson’s disease. An annotation of all 47 SNPs in our model can be found in the Supplementary Information. An exciting result from this analysis of the PPMI dataset is the statistical interaction of variants rs3822019 on chromosome four in gene *TMEM175*, coding for a potassium channel in late endosomes, and rs17022,452 on chromosome 2, close to the coding region of *GAPDHP25*, glyceraldehyde-3 phosphate dehydrogenase pseudogene 25. rs3822019 is an intron variant that has been linked to Parkinson’s disease. (Nalls et al., 2014).

## Discussion

We analyzed the PPMI dataset and built predictive models using polygenic risk scores, a deep learning algorithm for genomic data (Romero et al., 2016), and LASSO regression with and without interaction-based feature selection to reduce the hypothesis space. The PRS model comprises 57 SNPs and showed an AUC of 0.58 whereas the deep learning model had an AUC of 0.67. Notably, the deep learning model consists of abstract embeddings instead of single SNPs like the PRS. Therefore, identification of disease-associated SNPs and further insights into the disease mechanism are not possible here. The LASSO regression model built on interactions containing only 47 SNPs that were discovered via the use of contextual information outperformed the other predictive models with an AUC of 0.85. Beyond that, the approach was able to associate new variants with the disease that would not have shown up under an additive approach such as PRS.

We investigated how the combinations of the relevant genotypes rs3822019_TT (*TMEM175*) and rs17022,452_GG (*GAPDHP25*) split the individuals into cases and controls (Table 3). All subjects that are homozygous for rs3822019_TT are affected by PD. Furthermore, most individuals heterozygous for this variant (rs3822019_TT) or homozygous for rs17022,452_GG are cases (76.4 and 75.0%, respectively). These results support the relevance of the association between these variants and PD status.

**TABLE 3:** PD cases and controls among bearers of the respective genotype combinations of the identified variants rs3822019 and rs17022,452.

Genotype combination	Cases	Controls
rs3822019_TT/rs17022,452_GG	0	0
rs3822019_TT/rs17022,452_GA	6/100%	0
rs3822019_TC/rs17022,452_GG	2/50%	2/50%
rs3822019_TT/-	7/100%	0
-/rs17022,452_GG	7/87.5%	1/12.5%
rs3822019_TC/rs17022,452_GA	27/87.1%	4/12.9%
rs3822019_TC/-	68/73.9%	24/26.1%
-/rs17022,452_GA	66/75%	22/25%
-/-	113/56.5%	87/43.5%

The *TMEM175/GAK/DGKQ* locus was the third strongest risk locus in a GWA study of Parkinson’s disease (Krohn et al., 2020) and has been described as a potential drug target. (Diogo et al., 2018; Jinn et al., 2019). Deficiency in the potassium channel TMEM175 results in unstable lysosomal pH, which leads to decreased lysosomal catalytic activity and increased α-synuclein aggregation, among other effects. As a potassium channel, TMEM175 has a high potential as a druggable target and a tractable therapeutic strategy has been proposed. (Jinn et al., 2017).

GAPDH has been targeted with the investigational drug Omigapil for prevention of PD, ALS, congenital muscular dystrophy and myopathy. The drug has been shown to protect against behavioural abnormalities and neuro-degeneration in animal models of Parkinson’s disease. However, PD development has been terminated due to lack of benefit. (Olanow et al., 2006).

There seem to be various causes of Parkinson’s disease, yet the pathogenesis of this disease appears to be converging on common themes—oxidative stress, mitochondrial dysfunction, and protein aggregation—all of which are tightly linked to autophagy. (Lynch-Day et al., 2012). Both TMEM175 (Jinn et al., 2019) and GAPDH (Butera et al., 2019) regulate autophagy. Disturbed expression of autophagy genes in blood of PD patients. (Lynch-Day et al., 2012).

To summarize, we here present an approach to apply machine learning algorithms to high-dimensional genomic data using a contextual knowledge based feature selection. PRS models require a large set of SNPs, which leads to overfitting and limits their use in clinical practice. We generated more parsimonious models overcoming these limitations–with only 47, partly interacting SNPs, our model was able to outperform a PRS model based on 57 SNPs for Parkinson’s disease. Analysis of feature importance of our model identified a gene-gene interaction of *TMEM175* and *GAPDHP25*. TMEM175 has been described as a potential drug target and further information on its mechanism of action could be invaluable. A recently discovered interaction with pseudogene *GAPDHP25* could provide helpful insights. In conclusion, applying machine learning algorithms to feature-selected genomic data led to an interaction-based model with better predictive performance than PRS and has paved the way for the generation of new insights into disease mechanisms.

## Methods

### Parkinson’s Progression Marker Initiative Dataset

The Parkinson’s Progression Marker Initiative (PPMI) dataset (https://www.ppmi-info.org) contains 471 subjects (368 cases and 152 controls), and for each subject, genotyping information collected from two complementary chips (NeuroX and ImmunoChip) is available. (Marek et al., 2011). After careful quality control and harmonization (e.g., genome build conversion, strand alignment) as described in the literature (Marees et al., 2018), we merged that information into a single dataset with 380,939 variants in total.

After this initial data harmonization, an additional set of quality control steps were performed on variants and individuals that aimed to remove biases that could affect the downstream analysis. First, SNPs and individuals were filtered based on their missingness in the dataset. This ensured the exclusion of SNPs that had a high proportion of subjects where genotyping information was unavailable or of poor quality. Similarly, individuals where a large proportion of SNPs could not be measured were excluded. This step was achieved by setting the missing call rate threshold to 0.02 (i.e., >2%); as a result, 6,084 variants and 22 people were removed. SNP filtering was performed before individual filtering.

With high missing call rates filtered, all variants not on autosomal chromosomes were removed (5,731 variants). This was followed by the identification and removal of variants deviating from Hardy-Weinberg equilibrium, which can indicate genotyping errors. These variants were identified in a two-stage process whereby we first applied a threshold of 1e-6 exclusively to controls, followed by a threshold of 1e-10 applied to all samples, leading to the removal of 0 and 202 variants, respectively.

Next, individuals were filtered based on their heterozygosity rates, which can indicate sample contamination. Individuals deviating by more than 3 standard deviations from the mean of the rate of all samples (13 individuals) were removed. To assess the heterozygosity rate per sample, variants in linkage disequilibrium were first extracted, scanning the genome at a window size of 50 variants, a step size of 5, and a pairwise correlation threshold of 0.2.

Finally, relatedness between individuals was ascertained through the calculation and assessment of their respective identity by descent coefficients (IBD). Only one individual in a related pair would be kept, although in this case, no related individuals were identified and so none were removed.

The final quality-controlled dataset contained 369,036 variants and 436 individuals passing the various filters.

## Genome-Wide Association Study

As a preliminary step, a genome-wide association (GWA) analysis was performed with the R package SAIGE (Zhou et al., 2018) to test individual variants for their association with Parkinson’s disease.

### Polygenic Risk Score

The PRS was constructed by using PLINK (Purcell et al., 2007) following the guidelines provided by Choi et al. (Choi et al., 2020) and the accompanying tutorial (https://choishingwan.github.io/PRS-Tutorial/plink/). The clumping cut-off of *r* ^2^ was 0.1. For all subjects in the training, validation and test sets, seven distinct risk scores were calculated, corresponding to seven potential *p*-value thresholds (0.001, 0.05, 0.1, 0.2, 0.4, 0.5). The seven risk scores for the subjects in the training set were then used to train seven separate logistic regression classifiers (one for each *p*-value threshold) using the *glm* function in R (www.R-project.org). These classifiers were evaluated relative to the validation data set, leading to the selection of the classifier based on the PRS calculated using the *p*-value threshold of 0.05. The predictions of this final classifier were then evaluated relative to the test set.

### Deep Learning

The deep learning prediction model was built using a Diet Network according to the procedure described by Romero et al. (Romero et al., 2016) The model is composed of three networks: one basic and two auxiliary networks. After a basic discriminative network with optional reconstruction path, follows a network that predicts the input fat layer parameters, and finally, a network that predicts the reconstruction fat layer parameters. The official code can be found here: https://github.com/adri-romsor/DietNetworks.

### Feature Selection

The interaction-based feature selection approach that we adopt organizes data mined from journal articles, pathway libraries, protein co-expression libraries, and drug candidate libraries (e.g., dbSNP, ClinVar, OMIM, Reactome, STRING database) into a hierarchical knowledge graph, which generates disease-specific hypotheses based on interactions of genetic variants (Figure 1). Each interaction’s predictive power is determined using the training data set and the *glm* function in R (www.R-project.org). If an interaction predicts disease status well, the graph is incentivized to ‘fine-tune’ the hypothesis by comparing a set of very similar hypotheses. If a hypothesis has little or no predictive power, the graph is not incentivized to explore it or similar hypotheses further and will instead propose hypotheses containing different variants. (Klinger et al., 2021). This learning process is driven by gradient descent, meaning that it converges when the average performance of the new multi-variant hypothesis does not increase. After convergence, the selected features are used to build prediction models with standard machine learning algorithms, such as LASSO regression (Friedman et al., 2010).

### LASSO Regression

LASSO (least absolute shrinkage and selection operator) regression models were computed by using the glmnet package (https://glmnet.stanford.edu/index.html) for R (www.R-project.org) and its function cv.glmnet with five-fold cross-validation in order to avoid overfitting. (Friedman et al., 2010).

## Data Availability

Publicly available datasets were analyzed in this study. This data can be found here: https://www.ppmi-info.org/.
